# Psychology, Physical Activity, and Post-pandemic Health: An Embodied Perspective

**DOI:** 10.3389/fpsyg.2021.588931

**Published:** 2021-03-05

**Authors:** Haney Aguirre-Loaiza, Antonio Mejía-Bolaño, Juliana Cualdrón, Sarah Ospina

**Affiliations:** Department of Psychology, Universidad Católica de Pereira, Pereira, Colombia

**Keywords:** COVID-19, children, obesity, mental health–related quality of life, sedentary behavior, physical inactivity, exercise, cognition & emotion

## Introduction

The world changed after coronavirus disease 2019 (COVID-19) started spreading. Lockdowns and isolation amount to direct and indirect effects whose consequences are yet to be analyzed more deeply. These social measures affect the health of those who have not been infected (Carter et al., 2020). In this regard, experts and union organizations have recommended home physical activity (PA) (ACSM., 2020). PA can be a protective factor to deal with problems related to the pandemic. By the same token, technological resources and video tutorials are useful tools for PA at home. However, possible collateral effects of their use must be considered as well as the supervision of qualified professionals (Aguirre-Loaiza et al., 2019a; Papaioannou et al., 2020).

Due to the COVID-19 emergency, several states and institutions have closed and followed preventive measures, as suggested by the WHO (Mattioli et al., 2020). Considering that schools generally promote the development of motor skills and knowledge of basic PA for health purposes (e.g., physical education lessons), it is worrying that aspects such as food and sleep schedules have also been negatively impacted since they are directly related to several health problems, including the risk of obesity (Pietrobelli et al., 2020). Some studies indicate that eating and sleeping patterns changed negatively 3 weeks after the confinement started (Pietrobelli et al., 2020).

Academic communities and government agendas face serious post-pandemic challenges. Mainly, they will have to ensure an action plan to promote PA educational strategies for health purposes. Furthermore, this situation represents an opportunity to bring into question the mind–body dichotomy, which can open a space for embodied cognition (EC) and its wide conceptual spectrum to enable research development and applied knowledge of PA. We suggest that future research and its implication should be approached from the standpoint of EC and its possible effects on the cognitive processes associated with PA.

There is increasing evidence in favor of a positive association between PA and cognitive functions. Moderate-to-vigorous intensities are positively related to better memory performance (Berrios Aguayo et al., 2019) and selective attention (Chang et al., 2014). In addition, PA improves cognitive performance in dual tasks involving response inhibition (Joyce et al., 2014). In fact, acute exercise (~1 h) provides benefits in cognitive processes, e.g., attention, memory, problem solving, language, cognitive flexibility, and inhibitory control (Basso et al., 2015; Basso and Suzuki, 2017).

Neuroscientific experimental designs in animal models have also given clues that contribute to clarify the relationship between PA and cognitive functions. For example, it has been shown in rodents that the protein cathepsin B (CTSB), which is increased with PA, could affect brain tissue, and it has been demonstrated that if these proteins were applied to the progenitor cells of the hippocampus, they could have an impact on its neurogenesis process (Suzuki, 2016).

## Consequences of Coronavirus Disease 2019 on Physical Activity and Health

In general terms, COVID-19 has directly affected people's quality of life (Aguirre-Loaiza et al., 2020). Some of the signs and symptoms linked to social confinement are stress, fear, panic attacks, anxiety, and depression (Brooks et al., 2020; Rajkumar, 2020). Negative emotions reduce the quality of sleep (Zhang et al., 2020), and sleep disorders can become risk factors leading to suicidal behaviors (Sher, 2020). Similarly, alterations in PA and eating patterns have compromised people's health (Ammar et al., 2020). Infected patients have more serious respiratory complications when there is a high correlation between obesity and other illnesses (Petrilli et al., 2020).

PA has been affected by the social isolation measures that have been implemented. Some results point out that 1 week before the pandemic started, PA had an average duration of 540 min per week, but during the pandemic, it reduced to 105 min per week (Xiang et al., 2020). Likewise, there is PA reduction in students, while sedentary activities, calorie consumption, and exposure to electronic devices have increased (Arévalo et al., 2020; Rundle et al., 2020). As a whole, these factors represent a risk to develop main non-communicable diseases. Although guidelines have been suggested for children and adolescent PA (Radom-Aizik, 2020) and for their return to school environments (Chen et al., 2020), the near future might be discouraging due to a likely new wave of obesity and psychopathology cases. Consequently, scientific and government strategies must promote PA to alleviate several of the difficulties caused by COVID-19 (Zhu, 2020) and child obesity (King et al., 2020).

## Embodied Cognition and Physical Activity

Every day more evidence of the positive impact of PA in cognitive development is found (Mandolesi et al., 2018; Aguirre-Loaiza et al., 2019a; Doherty and Forés Miravalles, 2019; Erickson et al., 2019), which not only helps to the consolidation of knowledge on this area but also offers promising perspectives for multifactorial studies involving young students. Nevertheless, theoretical underpinnings have emphasized the mind–body dichotomy. EC proposes an inseparable relation of cognitive processes and body interactions, which creates a causal dependency on mental events that derive on body actions in a specific environment (Kiverstein, 2018).

The basic tenets of EC suggest that cognitive functions depend on their use and interaction with the environment (Shapiro and Spaulding, 2019). For example, children's play is an important event for the application of EC.

Although theories of cognitive development differ between innate and learned, there is a large part of them that support the link between perceptual, cognitive, and action skills. It is through the sensory-motor experience, that is, in contact with the world and objects, that babies build a representation of concepts about their physical environment and the objects that are present there (Gibbs, 2005).

In adults, coordination activities constitute a rich interaction between body and the environment. In both cases, action–perception patterns lead to adaptive coupling patterns (Cappuccio, 2019). Sensory experiences that arise when the body moves are responsible for building the foundations of learning through action and perception (Moreau and Tomporowski, 2019). In this representational scheme, cognitive functions (e.g., control, anticipation, perceptual discrimination, memory, and linguistic representations) are not stored in the brain, but they are functions modeled through the interaction with the environment (Cappuccio, 2019).

Currently, the notion that athletic performance might aid the understanding of the mind has been praised (Shapiro and Spaulding, 2019). Following this trend, PA and sport in general offer several possibilities to recognize that cognitive functions do not stand apart from the body. EC introduced new ways of understanding human and social interaction (Cappucio, 2019; Shapiro and Spaulding, 2019). Processes involving the body and its surroundings contribute to modeling the basic forms of cognition (sensory-motor, affective, and adapative) as well as the higher ones (inferential, linguistic, and representational) (Cappuccio, 2019). Consequently, it is possible to abandon the dualistic conception existing between cognition and PA. Neuronal processes have been reported to be healthier and more efficient after PA, leading to a regulation of the negative effects of stress on brain neurotrophic factors (Moreau and Tomporowski, 2019), which mediate many of the benefits that PA has on cognition.

The aforementioned is supported by enactive and extended theories. The former proposes that cognitive capacities arise from the adaptive interaction between subjects and their ecological environment (Cappuccio, 2019), whereas the latter suggests that the mind is integrated to its environment and that the environment is responsible for the representations that the mind has of its surroundings and their constant change. Likewise, body actions and the ecological environment in which subjects act can be foundational elements of a cognitive process (Kiverstein, 2018).

On the other hand, the integration of knowledge through motor actions has become increasingly important for learning processes. As an example, one recent study compared 2 learning groups: a sedentary one and another of embodied PA practice. The study showed positive results for the embodied group (Schmidt et al., 2019). The motivational aspects that an embodied approach entails have their roots in the embodiment thesis that hold cognition and affection as inseparable. Here, affective patterns are foundational to perception, action, and behavior (Kirchhoff, 2018). Learning environments are appropriate contexts in which PA and its cognitive benefits are promoted (Aguirre-Loaiza et al., 2019b). Therefore, the commitment and participation of school students should increase.

This can explain the role that PA has in cell generation since, in order to survive, neurons must come into contact, and this process improves when PA is interlinked with learning (Moreau and Tomporowski, 2019). We have illustrated situations that involve coordination and play processes, but yoga has also showed how to integrate EC (Rashedi and Schonert-Reichl, 2019). Additionally, dancing can also be promising for any population group. The synchronization of movement and music, the memorization of step sequences, and the interaction with the surroundings improve cognitive and physical functions through perception, execution, memory, and motor skills (Borhan et al., 2018). Thus, through EC, a theoretical framework for the integration of PA for health purposes and people's welfare can be justified.

Additionally, since clinical neuropsychology discovered through mirror neurons the relationship between language and the motor system, it was possible to explore neurodegenerative diseases in which the motor system and language are involved (Cardona, 2017), as well as processes of anticipation and comprehension (Amoruso et al., 2014).

## Future Research Opportunities and Courses of Action

PA is essential to maintain a stable health. By keeping an adequate physical condition, cognitive, and physical processes can be improved. Games and dancing are alternatives that can be considered for children. Meanwhile, coordination or dual activities that use the body are to be implemented.

Future research can be projected in two purposes: (a) to understand the interaction between PA and EC and its health and educational implications. It can be useful, in the midst of the COVID-19 contingency, to approach PA and EC alternatives as theoretical and methodological proposals, mainly in anticipation of post-pandemic psychopathology cases (Wade et al., 2020). (b) To reveal the role of athletic performance on cognitive functions. Here, sports psychology has a potential course of action to be explored (Cappuccio, 2019).

The experience of a pandemic and its economic, social, and health consequences suggest that measures based on scientific evidence are taken for granted, such as the impact of physical education on school children and their extracurricular life as well as the motivation to continue healthy lifestyle practices (Evans and Davies, 2010). In general, the challenge of future research is demanding, and it is a matter of furthering the understanding of the link between body and cognition in the face of which educational institutions, families, and government agendas must strive to offer such opportunities.

In regard to short-term action—and considering that after the COVID-19 pandemic educational contexts will highly depend on virtual tools and that, therefore, this will result in sedentary behavior—educational policies should look for ways to integrate the body activities to subjects such as mathematics, biology, and others. However, educational contexts should also make health care a priority using EC schemes starting from early ages.

## Author Contributions

HA-L, AM-B, JC, and SO contributed in writing, reviewing, and editing of the manuscript. All authors contributed to the article and approved the submitted version.

## Conflict of Interest

The authors declare that the research was conducted in the absence of any commercial or financial relationships that could be construed as a potential conflict of interest.
